# A novel *LMNA* mutation identified in a Japanese patient with LMNA-associated congenital muscular dystrophy

**DOI:** 10.1038/s41439-018-0018-6

**Published:** 2018-07-20

**Authors:** Akihiko Ishiyama, Aritoshi Iida, Shinichiro Hayashi, Hirofumi Komaki, Masayuki Sasaki, Ikuya Nonaka, Satoru Noguchi, Ichizo Nishino

**Affiliations:** 1 Department of Child Neurology, National Center Hospital, National Center of Neurology and Psychiatry, Tokyo, Japan; 2 Department of Neuromuscular Research, National Institute of Neuroscience, National Center of Neurology and Psychiatry, Tokyo, Japan; 3 Department of Clinical Genome Analysis, Medical Genome Center, National Center of Neurology and Psychiatry, Tokyo, Japan

## Abstract

LMNA-associated congenital muscular dystrophy (L-CMD) is a severe form of muscle laminopathy. *LMNA* encodes lamin A, which an intermediate filament protein that attaches to the inner membrane of the nuclear envelope. We performed sequence analysis based on our original targeted gene panel system for muscle diseases to obtain a molecular diagnosis in a Japanese girl with L-CMD. A novel heterozygous missense mutation, c.115A>C (p.Asn39His), in *LMNA* is reported.

Laminopathies are a heterogeneous group of disorders caused by pathogenic variants in the lamin A genes (*LMNAs*)^1,2^. More than 13 different hereditary disorders have been recognized^3^. Among these, three are LMNA-related muscular dystrophies, including autosomal dominant Emery-Dreifuss muscular dystrophy^4^, limb-girdle muscular dystrophy type 1B, and LMNA-associated congenital muscular dystrophy (L-CMD). In L-CMD, the initial symptom is usually decreased fetal or newborn movements followed by a significant delay in motor development^5^. Muscle weakness is initially predominant in the cervical-axial muscles of the proximal muscles. A drooping head or poor head control due to neck muscle weakness is characteristically observed in some patients^6^. Cardiac involvement and joint contracture can also be seen in some patients. Pathologically, there may be marked inflammatory changes, such as perivascular cuffing and endomysial/perimysial lymphocyte infiltration^7^. L-CMD is due to de novo genetic variations in exon 1 and exon 6 of *LMNA*, which encode its coil 1A and coil 2 domains, respectively^5^. Interestingly, four types of amino acid substitutions at Asn39 in the coil 1A domain have been reported in L-CMD patients^5–9^. In this study, we report a Japanese girl with L-CMD who harbored the novel heterozygous missense mutation c.115A>C (p.Asn39His) in *LMNA*.

The National Center of Neurology and Psychiatry (NCNP) is a referral center for neuromuscular diseases in Japan. Since 1978, we have made pathological diagnoses based on more than 18,000 muscle biopsies. For cases with undiagnosed hereditary muscle disease, we now perform mutation screening using an Ion PGM sequencer (Thermo Fisher Scientific, MA, USA) in combination with our recently developed targeted gene panels, which cover 187 genes known to cause hereditary muscle diseases. We developed four such panels: muscular dystrophy (MD panel), congenital myopathy, metabolic myopathy, and myopathy with protein aggregations/rimmed vacuoles, respectively^10^.

The patient was a girl aged 3 years and 6 months old at the time of muscle biopsy. She had no family history of neuromuscular disease. She was born to healthy parents without asphyxia by normal delivery at 35 weeks of gestation. Her height, weight, and head circumference at birth were 44 cm, 2150 g, and 31.0 cm, respectively. She could hold her head up at 4 months, could take a sitting position at 1 year, and could walk independently at 18 months, indicating a motor developmental delay. There was no delay in intellectual development. When she was 3 years old, a kindergarten teacher noted that she was a slow runner and fell easily. She was referred to our hospital because of motor developmental delay. At presentation, her height and weight were 89.2 cm (−1.6 SD) and 11.8 kg (−1.4 SD), respectively. Ankle contracture was evident. She used the Gowers’ maneuver to stand up and used the railing to go upstairs. Blood tests revealed high levels of the following serum enzymes: AST, 60 U/L; ALT, 63 U/L; CK, 1673 U/L; and aldolase, 21.5 U/L (2.1–6.1). There were no abnormalities in nerve conduction studies, but electromyography disclosed discharge at rest, suggesting a myogenic disease, including muscular dystrophy. Hematoxylin eosin staining of muscle biopsy specimens obtained from the biceps brachii showed scattered necrotic and regenerating fibers as well as marked variations in fiber size and marked endomysial fibrosis, suggesting chronic necrotic and regenerating processes compatible with muscular dystrophy (Fig. 1a). There was no lymphocyte infiltration. Immunohistochemical staining showed that muscle fibers were positive for MHC class I (Fig. 1b). Type 2C fibers accounted for 28%. No biological samples were available from the parents.

**Fig. 1:**
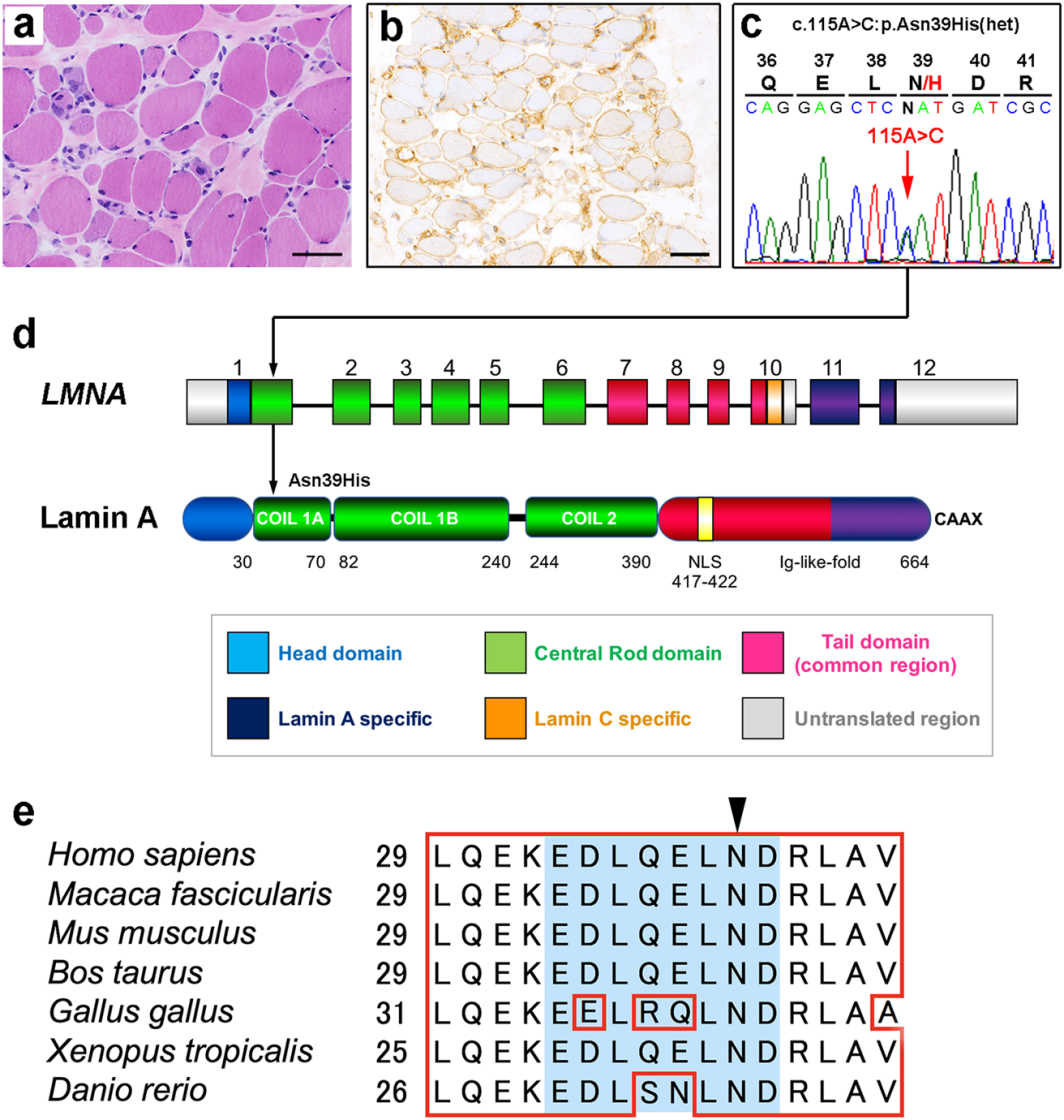
A novel missense mutation in *LMNA* causes LMNA-associated congenital muscular dystrophy. **a** Muscle biopsy tissue sections from patients showing the histological features of muscular dystrophy. Bar: 50 µm. **b** Immunostaining for MHC class I in patient muscle sections. The sarcolemma and cytoplasm of non-necrotic muscle fibers are shown stained. Bar: 50 µm. **c** Sanger sequencing analysis of the patient. A novel missense heterozygous mutation was identified: c.115A>C (p.Asn39His) in exon 1 of *LMNA*. **d** Schematic of *LMNA* exons and corresponding protein domains. The location of the mutation identified in this study is indicated by a black arrow. **e** Alignment of the lamin A amino acid sequence in different species. The open red box indicates identical amino acids among all species. The blue box indicates acidic patches. The arrowhead indicates the position of the p.Asn39His mutation

Clinical information and materials were obtained from the patient for diagnostic purposes, and they were approved for scientific use via written informed consent. All experiments in this study were approved by the Ethical Committee of NCNP.

To evaluate the possibility of the patient being a dystrophinopathy carrier, a multiplex ligation-dependent probe amplification assay for *DMD* was performed but found to be negative. We then ran an Ion PGM sequencer in combination with the MD targeted gene panel. A total of 204 Mb sequences were obtained and mapped to the human RefSeq (GRchg37/hg19). At least 97.6% of the target gene loci were covered at a depth of 20 reads. A novel heterozygous missense mutation, c.115A>C (p.Asn39His), was detected in *LMNA* (Fig. 1c). We confirmed this mutation by direct sequencing of PCR products obtained from the DNA of the patient. The mutation was located within the 1A domain of the central rod domain, which has been implicated in dimerization in lamins (Fig. 1d). The asparagine caused by the mutation is highly conserved among species (Fig. 1e). The mutation was not deposited in any databases, including dbSNP, 1000 genomes, the Human Gene Mutation Database, the Exome Aggregation Consortium, the Human Genetic Variation Database, the Integrated Japanese Genome Variation Database or ClinVar (as of the end of February, 2018). Three mutation-prediction tools were also used to check the functional effects of this substitution. SIFT, PolyPhen-2 and Mutation Taster predicted that it would be “deleterious” with a score of 0.001, “probably damaging” with a score of 0.999 and “disease-causing” with a score of 1, respectively.

In this study, we identified the p.Asn39His mutation in a Japanese girl with L-CMD. As mentioned above, four types of missense mutations at the same site, Asn39Ser, Asn39Asp, Asn39Tyr, and Asn39Lys, have previously been reported in L-CMD patients^5–9^, even though Asn39Ser variants are also associated with EDMD^9^. Quijano-Roy et al. classified L-CMD patients into two groups: the first have a severe congenital muscular dystrophy phenotype, and the second have a dropped-head syndrome phenotype^5^. When we compared the clinical features of our patient (Asn39His) to those reported in patients with Asn39Ser, Asn39Asp, Asn39Tyr, or Asn39Lys mutations, some similarities and differences in phenotypes emerged among the patients (Table 1). There was a muscle weakness pattern in which the axial and proximal muscles were predominantly affected in patients with all mutations; dropped head was observed in the patients with Asn39Ser, Asn39Tyr, and Asn39Lys but not in those with Asn39Asp and Asn39His; serum CK level, moderate to high increase in all mutations; joint contractures were positive in all mutations; cardiac involvement was positive in Asn39Asp, Asn39Lys and 1 case of Asn39Ser but negative in Asn39Tyr, Asn39His and 2 cases of Asn39Ser; and inflammation in muscle pathology was positive in Asn39Asp and Asn39His but negative in the other mutations. Although these differences may be affected by the age of the patient or the disease duration, substitutions at different amino acids in the same site may influence the structure as well as the function of the nuclear lamin A matrix. Various types of amino acid substitutions observed at the same site must be explored as they provide precious information that increases our understanding of the rather complicated phenotype-genotype correlations observed in LMNA-related myopathies.

**Table1:** Clinical information of the patients with LMNA mutation at position Asn39

Patient # in ref. /sex	Amino acid substitution	Initial signs (age)	Axial and proximal muscle weakness	Dropped head (age)	CK level	Joint contractures	Cardiac involvement	Inflammation in pathology	Reference #
-/F	Asn39His	Motor developmental delay (1 year)	Yes	No	1673 (IU/L)	Ankles	No	Lymphocyte infiltration; No (MHC class I positive)	This study
11/F	Asn39Ser	Hypotonia, dropped head (4 mo)	Yes	Yes	X4^a^	Early: ankles Late: hips, elbows	No	No description	Quijano-Roy et al.^5^
3/F	Asn39Ser	Motor developmental delay, poor head control (5–6 mo)	Yes	Yes	X4^a^	Ankles, knees, hips, elbows, wrists, hands, and fingers	No	No description	Pasqualin et al.^8^
4/M	Asn39Ser	Poor head control (5–6 mo)	Yes	Yes	X2^a^	Elbows, hips, knees, and ankles Distal upper-limb hyperextensibility (3 year)	Frequent ventricular extrasystoles	No description	Pasqualin et al.^8^
3/M	Asn39Asp	Motor developmental delay (11 mo)	Yes	No	1100 (IU/L)	Ankles, knees, hips, Rigid spine from childhood	13 year: 200B0 A-V block, 15 year: 3˚ A-V block, pacemaker implantation	Lymphocyte infiltration; moderate (MHC class I positive)	Komaki et al.^6^
-/M	Asn39Tyr	Hypotonia, motor developmental delay (5 mo)	Yes	Yes (5 years)	553–1050 (IU/L)	Early: distal Late: proxmal limbs, elbow, spinal stifness with dorsolumbar hyperlordosis	No	Not observed	Prigogine et al.^7^
6/F	Asn39Lys	Proximal weakness, dropped head (4 mo)	Yes	Yes	906–1062 (IU/L)	Lordosis	Sinus tachycardia	Not observed	Tan et al.^9^

*CK* Creatine kinase

^a^Increase of serum CK level above the normal upper limit value

Asn at 39, which is located at the 8th residue from the *N*-terminus in the coil 1A domain, is highly conserved during evolution (Fig. 1e). This residue is also conserved at more than 98% among various intermediate filament members (type I–V), in which lamin A belongs to type V. Comprehensive sequence comparisons of the coil 1A segment among all types of intermediate filaments and the crystal structure of vimentin have suggested that segment coil 1A has unique features that distinguish it from the other major coiled-coil segments^11^. Special structural roles of coil 1A have been predicted because it can form a rigid coiled-coil structure at its hydrophobic C-terminal region and an open structure at its hydrophilic *N*-terminal region, and these function to unwind the molecules into separate a-helix strands (monomers) and to then reassemble them into a coiled-coil rope (dimes) under appropriate conditions^12^. This Asn residue may provide the *N*-terminal region with hydrophilic properties that regulate these structural changes.

More interestingly, the region including Asn at 39 in coil 1A was predicted to function in potential head-to-tail association interactions in nuclear lamins^12^. The coil 1A and coil 2 dimers each have one pronounced patch of negative electrostatic potential: EDLQELND (residue 33–40, net charge −4 in coil 1A) and DEYQELLD (residue 357–364, net charge −4 in coil 2), respectively. These acidic patches in coil 1A and coil 2 may be electrostatically attracted by the positively charged arginine clusters in the Tail and Head domains, respectively. Thus, changes at the His and Lys at 39 in L-CMD will give a positive charge to N-terminal acidic patch, resulting in an increased net charge of −3, which weakens the head-to-tail association of lamin A dimers and leads to unstable network formation. In contrast, the change to Asn at 39 (resulting in a decreased net charge of −5) will have the opposite effect. The pathogenic variant p.Glu358Lys in coil 2 in L-CMD may support this idea. Further analyses of the structural changes in and functional roles of the mutated LMNA proteins will be required to determine their effects on the pathogenesis of L-CMD.

## Data Availability

The relevant data from this Data Report are hosted at the Human Genome Variation Database at 10.6084/m9.figshare.hgv.2342.
